# Tetra­aqua­bis­[2-(2-oxo-2,3-dihydro-1,3-benzoxazol-3-yl)acetato]­zinc

**DOI:** 10.1107/S1600536811007999

**Published:** 2011-03-12

**Authors:** Jamshid Ashurov, Gavhar Karimova, Nasir Mukhamedov, Nusrat A. Parpiev, Bakhtijar Ibragimov

**Affiliations:** a Institute of Bioorganic Chemistry, Academy of Sciences of Uzbekistan, Mirzo Ulugbek Str. 83, Tashkent 100125, Uzbekistan; b The National University of Uzbekistan named after Mirzo Ulugbek, Faculty of Chemistry, University Str. 6, Tashkent 100779, Uzbekistan; c S. Yunusov Institute of the Chemistry of Plant Substances, Academy of Sciences of Uzbekistan, Mirzo Ulugbek Str. 77, Tashkent 100170, Uzbekistan

## Abstract

The Zn^II^ ion in the title compound, [Zn(C_9_H_6_NO_4_)_2_(H_2_O)_4_], is located on an inversion center and is octa­hedrally coordinated by two 2-(2-oxo-2,3-dihydro-1,3-benzoxazol-3-yl)acetate anions in axial sites and four water mol­ecules in equatorial positions. In the crystal, O—H⋯O hydrogen bonds between the coordinated water mol­ecules and carbon­yl–carboxyl­ate O atoms lead to pleated sheets parallel to (001).

## Related literature

For the synthesis of 3-alkanoic acid derivatives of 2(3*H*)-benzoxazolone, see: Lespagnol *et al.* (1967 ▶). For the biological activity of 2(3*H*)-benzoxazolone derivatives, see: Önkol *et al.* (2004 ▶). For the structure of a 2(3*H*)-benzoxazolone metal complex, see: Wagler & Hill (2008 ▶).
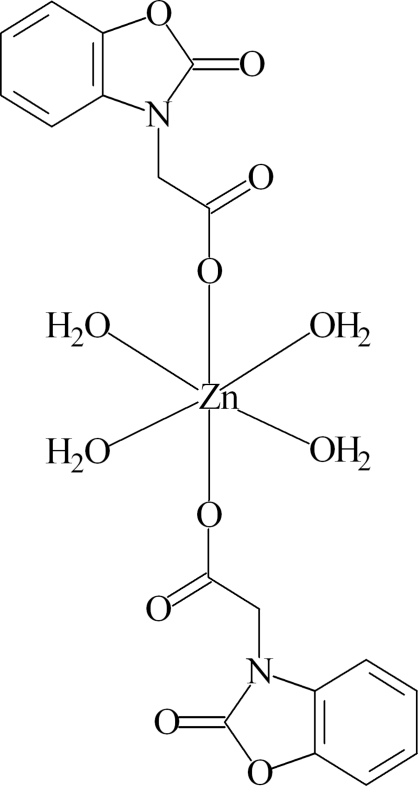


## Experimental

### 

#### Crystal data

[Zn(C_9_H_6_NO_4_)_2_(H_2_O)_4_]
*M* *_r_* = 521.73
Monoclinic, 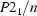

*a* = 6.144 (3) Å
*b* = 5.342 (1) Å
*c* = 30.595 (2) Å
β = 94.80 (5)°
*V* = 1000.6 (6) Å^3^
*Z* = 2
Cu *K*α radiation
μ = 2.38 mm^−1^
*T* = 293 K
0.50 × 0.35 × 0.20 mm

#### Data collection

Oxford Diffraction Xcalibur Ruby diffractometer
Absorption correction: multi-scan (*CrysAlis PRO*; Oxford Diffraction, 2009 ▶) *T*_min_ = 0.726, *T*_max_ = 1.000
5344 measured reflections
1745 independent reflections
1168 reflections with *I* > 2σ(*I*)
*R*_int_ = 0.065

#### Refinement

*R*[*F* ^2^ > 2σ(*F* ^2^)] = 0.053
*wR*(*F* ^2^) = 0.150
*S* = 1.06
1745 reflections
151 parameters
H-atom parameters constrained
Δρ_max_ = 0.80 e Å^−3^
Δρ_min_ = −0.34 e Å^−3^

### 

Data collection: *CrysAlis PRO* (Oxford Diffraction, 2009 ▶); cell refinement: *CrysAlis PRO*; data reduction: *CrysAlis PRO*; program(s) used to solve structure: *SHELXS97* (Sheldrick, 2008 ▶); program(s) used to refine structure: *SHELXL97* (Sheldrick, 2008 ▶); molecular graphics: *XP* (Bruker, 1998 ▶); software used to prepare material for publication: *SHELXL97*.

## Supplementary Material

Crystal structure: contains datablocks I, global. DOI: 10.1107/S1600536811007999/mw2002sup1.cif

Structure factors: contains datablocks I. DOI: 10.1107/S1600536811007999/mw2002Isup2.hkl

Additional supplementary materials:  crystallographic information; 3D view; checkCIF report

## Figures and Tables

**Table 1:** Hydrogen-bond geometry (Å, °)

*D*—H⋯*A*	*D*—H	H⋯*A*	*D*⋯*A*	*D*—H⋯*A*
O2*W*—H2*B*⋯O4^i^	0.83	2.00	2.772 (5)	156
O1*W*—H1*B*⋯O3^ii^	0.84	1.92	2.699 (5)	153
O1*W*—H1*A*⋯O2^iii^	0.82	2.07	2.799 (5)	148

Symmetry codes: (i) 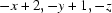; (ii) 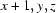; (iii) 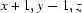.

**Fig. 1.**
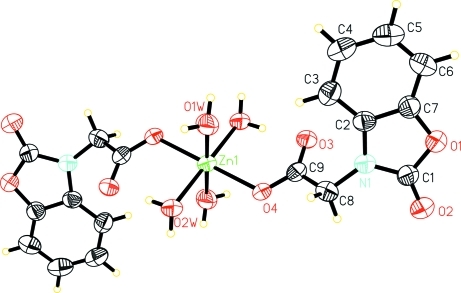
Molecular structure of the title compound with 50% probability displacement ellipsoids for non-H atoms.

**Fig. 2.**
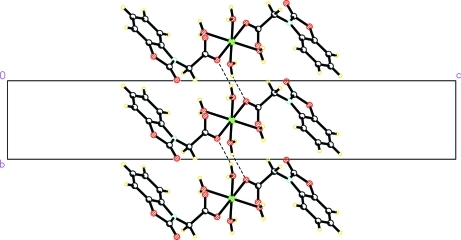
Part of the crystal structure of (I) projected down the a axis showing the formation of hydrogen bonded pleated strands along [010].

**Fig. 3.**
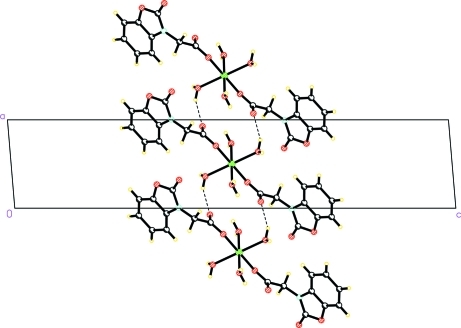
Part of the crystal structure of (I) projected down the b axis showing the formation of hydrogen bonded pleated strands along [100].

## supplementary crystallographic information

### Comment

2-Benzoxazolinone derivatives have attracted interest because of their biological activities (Önkol *et al.*, 2004).

The Zn (II) ion lies on an inversion center in an octahedral coordination environment with four O atoms from four coordinated water molecules in the equatorial positions and two O atoms from two ligands in the axial sites.(Fig.1). The coordinated water molecules form strong intermolecular hydrogen bonds with carbonyl and carboxyl O atoms of the ligand (Table 1). Centrosymmetric pairs of O2W–H2B···O4 hydrogen bonds propagating along [010] form pleated strands (Fig.2). Similar strands propagating along [100] (Fig.3) and [110] are formed by  O1W–H1B···O3 and O1W–H1A···O2 hydrogen bonds, respectively. Together, these interactions generate sheets parallel to (001).

### Experimental

A solution of 2-benzoxazolinon-3-yl-acetate acid (19,3 mg, 0.1 mmol) in ethanol (2 ml) was added to a solution of ZnCl_2_.6H_2_O (6.8 mg 0.05 mmol) in water (1 ml) and stirred for 10 min at 40 °C. Slow evaporation of the resulting solution gave colourles crystals suitable for X-ray analysis.

### Refinement

H-atoms attached to carbon were placed in calculated positions (C—H = 0.95 - 0.98 Å) while those attached to oxygen were placed in locations derived from a difference map and their positions adjusted to provide reasonable geometries for the coordinated water molecules. All were included as riding contributions with isotropic displacement parameters 1.2 - 1.5 times those of the attached atoms.

### Crystal data

**Table:**

[Zn(C_9_H_6_NO_4_)_2_(H_2_O)_4_]	*F*(000) = 536
*M**_r_* = 521.73	*D*_x_ = 1.732 Mg m^−^^3^
Monoclinic, *P*2_1_/*n*	Cu *K*α radiation, λ = 1.54180 Å
Hall symbol: -P 2yn	Cell parameters from 4608 reflections
*a* = 6.144 (3) Å	θ = 7.6–66.2°
*b* = 5.342 (1) Å	µ = 2.38 mm^−^^1^
*c* = 30.595 (2) Å	*T* = 293 K
β = 94.80 (5)°	Prism, colourless
*V* = 1000.6 (6) Å^3^	0.50 × 0.35 × 0.20 mm
*Z* = 2	

### Data collection

**Table:**

Oxford Diffraction Xcalibur Ruby diffractometer	1745 independent reflections
Radiation source: Enhance (Cu) X-ray Source	1168 reflections with *I* > 2σ(*I*)
graphite	*R*_int_ = 0.065
Detector resolution: 10.2576 pixels mm^-1^	θ_max_ = 66.7°, θ_min_ = 5.8°
ω scans	*h* = −7→6
Absorption correction: multi-scan (*CrysAlis PRO*; Oxford Diffraction, 2009)	*k* = −3→6
*T*_min_ = 0.726, *T*_max_ = 1.000	*l* = −35→36
5344 measured reflections	

### Refinement

**Table:**

Refinement on *F*^2^	Primary atom site location: structure-invariant direct methods
Least-squares matrix: full	Secondary atom site location: difference Fourier map
*R*[*F*^2^ > 2σ(*F*^2^)] = 0.053	Hydrogen site location: inferred from neighbouring sites
*wR*(*F*^2^) = 0.150	H-atom parameters constrained
*S* = 1.06	*w* = 1/[σ^2^(*F*_o_^2^) + (0.0732*P*)^2^ + 0.5645*P*] where *P* = (*F*_o_^2^ + 2*F*_c_^2^)/3
1745 reflections	(Δ/σ)_max_ < 0.001
151 parameters	Δρ_max_ = 0.80 e Å^−^^3^
0 restraints	Δρ_min_ = −0.34 e Å^−^^3^

### Special details

**Table:**

Geometry. All e.s.d.'s (except the e.s.d. in the dihedral angle between two l.s. planes) are estimated using the full covariance matrix. The cell e.s.d.'s are taken into account individually in the estimation of e.s.d.'s in distances, angles and torsion angles; correlations between e.s.d.'s in cell parameters are only used when they are defined by crystal symmetry. An approximate (isotropic) treatment of cell e.s.d.'s is used for estimating e.s.d.'s involving l.s. planes.
Refinement. Refinement of F^2^ against ALL reflections. The weighted R-factor wR and goodness of fit S are based on F^2^, conventional R-factors R are based on F, with F set to zero for negative F^2^. The threshold expression of F^2^ > 2sigma(F^2^) is used only for calculating R-factors(gt) etc. and is not relevant to the choice of reflections for refinement. R-factors based on F^2^ are statistically about twice as large as those based on F, and R- factors based on ALL data will be even larger. H-atoms attached to carbon were placed in calculated positions (C—H = 0.95 - 0.98 Å) while those attached to oxygen were placed in locations derived from a difference map and their positions adjusted to provide reasonable geometries for the coordinated water molecules. All were included as riding contributions with isotropic displacement parameters 1.2 - 1.5 times those of the attached atoms.

### Fractional atomic coordinates and isotropic or equivalent isotropic displacement parameters (Å^2^)

**Table:**

	*x*	*y*	*z*	*U*_iso_*/*U*_eq_	
Zn1	1.0000	0.0000	0.0000	0.0416 (3)	
O1	0.2330 (6)	0.1894 (7)	0.17363 (12)	0.0481 (9)	
O2	0.1804 (6)	0.4884 (7)	0.12215 (13)	0.0578 (10)	
O3	0.5737 (6)	−0.0487 (6)	0.05893 (11)	0.0432 (9)	
O4	0.7938 (6)	0.2347 (6)	0.03233 (11)	0.0446 (9)	
N1	0.4944 (7)	0.2399 (8)	0.12870 (14)	0.0427 (10)	
C1	0.2944 (9)	0.3231 (10)	0.13871 (17)	0.0448 (13)	
C2	0.5608 (8)	0.0474 (9)	0.15703 (16)	0.0386 (12)	
C3	0.7425 (9)	−0.1051 (10)	0.16032 (18)	0.0469 (14)	
H3A	0.8541	−0.0866	0.1418	0.056*	
C4	0.7500 (10)	−0.2885 (11)	0.19291 (19)	0.0563 (15)	
H4A	0.8694	−0.3959	0.1962	0.068*	
C5	0.5830 (10)	−0.3139 (11)	0.22046 (19)	0.0599 (16)	
H5A	0.5939	−0.4364	0.2421	0.072*	
C6	0.4014 (10)	−0.1616 (11)	0.21642 (18)	0.0547 (15)	
H6A	0.2885	−0.1791	0.2346	0.066*	
C7	0.3957 (8)	0.0144 (10)	0.18462 (16)	0.0438 (12)	
C8	0.6179 (9)	0.3428 (10)	0.09484 (17)	0.0479 (14)	
H8A	0.7561	0.4046	0.1083	0.057*	
H8B	0.5385	0.4845	0.0816	0.057*	
C9	0.6627 (8)	0.1603 (10)	0.05923 (16)	0.0397 (12)	
O1W	1.1399 (6)	−0.1149 (7)	0.06222 (12)	0.0539 (10)	
H1A	1.1269	−0.2588	0.0711	0.065*	
H1B	1.2722	−0.0835	0.0699	0.065*	
O2W	1.2325 (5)	0.2871 (6)	0.00289 (11)	0.0469 (9)	
H2A	1.3514	0.2751	−0.0076	0.056*	
H2B	1.2014	0.4359	−0.0014	0.056*	

### Atomic displacement parameters (Å^2^)

**Table:**

	*U*^11^	*U*^22^	*U*^33^	*U*^12^	*U*^13^	*U*^23^
Zn1	0.0426 (6)	0.0316 (5)	0.0524 (6)	0.0037 (5)	0.0147 (4)	0.0050 (5)
O1	0.046 (2)	0.045 (2)	0.056 (2)	0.0049 (18)	0.0188 (17)	0.0053 (18)
O2	0.059 (2)	0.050 (2)	0.067 (3)	0.016 (2)	0.0170 (19)	0.014 (2)
O3	0.045 (2)	0.036 (2)	0.050 (2)	−0.0054 (16)	0.0131 (16)	0.0012 (16)
O4	0.052 (2)	0.032 (2)	0.053 (2)	0.0059 (16)	0.0225 (17)	0.0060 (17)
N1	0.049 (3)	0.038 (3)	0.043 (2)	0.000 (2)	0.0105 (19)	0.001 (2)
C1	0.043 (3)	0.044 (3)	0.048 (3)	0.002 (3)	0.009 (2)	−0.003 (3)
C2	0.045 (3)	0.032 (3)	0.039 (3)	−0.002 (2)	0.006 (2)	−0.002 (2)
C3	0.042 (3)	0.044 (3)	0.055 (4)	0.001 (2)	0.003 (2)	−0.004 (3)
C4	0.058 (4)	0.047 (4)	0.062 (4)	0.006 (3)	−0.010 (3)	−0.006 (3)
C5	0.080 (5)	0.044 (4)	0.054 (4)	0.001 (3)	−0.006 (3)	0.006 (3)
C6	0.065 (4)	0.050 (4)	0.050 (4)	−0.009 (3)	0.009 (3)	0.010 (3)
C7	0.051 (3)	0.040 (3)	0.041 (3)	0.004 (3)	0.009 (2)	−0.004 (3)
C8	0.050 (3)	0.041 (3)	0.054 (3)	−0.006 (3)	0.016 (3)	0.006 (3)
C9	0.040 (3)	0.043 (3)	0.037 (3)	0.009 (2)	0.006 (2)	0.007 (2)
O1W	0.041 (2)	0.055 (2)	0.066 (3)	0.0016 (18)	0.0051 (18)	0.016 (2)
O2W	0.048 (2)	0.0305 (19)	0.064 (2)	0.0012 (16)	0.0170 (17)	0.0037 (17)

### Geometric parameters (Å, °)

**Table:**

Zn1—O4^i^	2.089 (3)	C3—C4	1.396 (8)
Zn1—O4	2.089 (3)	C3—H3A	0.9300
Zn1—O2W	2.093 (3)	C4—C5	1.388 (8)
Zn1—O2W^i^	2.093 (3)	C4—H4A	0.9300
Zn1—O1W	2.113 (4)	C5—C6	1.378 (8)
Zn1—O1W^i^	2.113 (4)	C5—H5A	0.9300
O1—C1	1.364 (6)	C6—C7	1.351 (8)
O1—C7	1.389 (6)	C6—H6A	0.9300
O2—C1	1.212 (6)	C8—C9	1.504 (7)
O3—C9	1.243 (6)	C8—H8A	0.9700
O4—C9	1.263 (6)	C8—H8B	0.9700
N1—C1	1.365 (6)	O1W—H1A	0.8218
N1—C2	1.384 (6)	O1W—H1B	0.8439
N1—C8	1.443 (6)	O2W—H2A	0.8249
C2—C3	1.379 (7)	O2W—H2B	0.8256
C2—C7	1.384 (7)		
			
O4^i^—Zn1—O4	180.00 (15)	C4—C3—H3A	121.8
O4^i^—Zn1—O2W	91.20 (13)	C5—C4—C3	121.4 (6)
O4—Zn1—O2W	88.81 (13)	C5—C4—H4A	119.3
O4^i^—Zn1—O2W^i^	88.80 (13)	C3—C4—H4A	119.3
O4—Zn1—O2W^i^	91.20 (13)	C6—C5—C4	121.5 (6)
O2W—Zn1—O2W^i^	180.0	C6—C5—H5A	119.3
O4^i^—Zn1—O1W	92.02 (14)	C4—C5—H5A	119.3
O4—Zn1—O1W	87.98 (14)	C7—C6—C5	116.6 (6)
O2W—Zn1—O1W	87.14 (14)	C7—C6—H6A	121.7
O2W^i^—Zn1—O1W	92.86 (14)	C5—C6—H6A	121.7
O4^i^—Zn1—O1W^i^	87.98 (14)	C6—C7—C2	123.5 (5)
O4—Zn1—O1W^i^	92.02 (14)	C6—C7—O1	128.1 (5)
O2W—Zn1—O1W^i^	92.86 (14)	C2—C7—O1	108.4 (4)
O2W^i^—Zn1—O1W^i^	87.14 (14)	N1—C8—C9	114.4 (4)
O1W—Zn1—O1W^i^	180.0	N1—C8—H8A	108.7
C1—O1—C7	107.6 (4)	C9—C8—H8A	108.7
C9—O4—Zn1	124.4 (3)	N1—C8—H8B	108.7
C1—N1—C2	109.0 (4)	C9—C8—H8B	108.7
C1—N1—C8	125.0 (4)	H8A—C8—H8B	107.6
C2—N1—C8	126.0 (4)	O3—C9—O4	125.6 (5)
O2—C1—O1	121.4 (5)	O3—C9—C8	118.7 (4)
O2—C1—N1	130.0 (5)	O4—C9—C8	115.7 (5)
O1—C1—N1	108.5 (4)	Zn1—O1W—H1A	121.8
C3—C2—N1	132.8 (5)	Zn1—O1W—H1B	120.1
C3—C2—C7	120.7 (5)	H1A—O1W—H1B	102.2
N1—C2—C7	106.5 (4)	Zn1—O2W—H2A	123.4
C2—C3—C4	116.4 (5)	Zn1—O2W—H2B	123.4
C2—C3—H3A	121.8	H2A—O2W—H2B	102.3

Symmetry codes: (i) −*x*+2, −*y*, −*z*.

### Hydrogen-bond geometry (Å, °)

**Table:**

*D*—H···*A*	*D*—H	H···*A*	*D*···*A*	*D*—H···*A*
O2W—H2B···O4^ii^	0.83	2.00	2.772 (5)	156
O1W—H1B···O3^iii^	0.84	1.92	2.699 (5)	153
O1W—H1A···O2^iv^	0.82	2.07	2.799 (5)	148

Symmetry codes: (ii) −*x*+2, −*y*+1, −*z*; (iii) *x*+1, *y*, *z*; (iv) *x*+1, *y*−1, *z*.
